# Telocytes and stem cells in limbus and uvea of mouse eye

**DOI:** 10.1111/jcmm.12111

**Published:** 2013-08-29

**Authors:** María José Luesma, Mihaela Gherghiceanu, Laurenţiu M Popescu

**Affiliations:** a Department of Human Anatomy and Histology Faculty of Medicine, University of ZaragozaZaragoza, Spain; b Aragon Health Research Institute (IIS Aragón)Zaragoza, Spain; c Laboratory of Electron Microscopy, ‘Victor Babeş’ National Institute of PathologyBucharest, Romania; d Department of Cellular and Molecular Medicine, ‘Carol Davila’ University of MedicineBucharest, Romania; e Division of Advanced Studies, ‘Victor Babeş’ National Institute of PathologyBucharest, Romania

**Keywords:** telocytes, stem cells, limbus, iris, cornea, cell junctions, exosomes, eye regeneration

## Abstract

The potential of stem cell (SC) therapies for eye diseases is well-recognized. However, the results remain only encouraging as little is known about the mechanisms responsible for eye renewal, regeneration and/or repair. Therefore, it is critical to gain knowledge about the specific tissue environment (niches) where the stem/progenitor cells reside in eye. A new type of interstitial cell–telocyte (TC) (http://www.telocytes.com) was recently identified by electron microscopy (EM). TCs have very long (tens of micrometres) and thin (below 200 nm) prolongations named telopodes (Tp) that form heterocellular networks in which SCs are embedded. We found TCs by EM and electron tomography in sclera, limbus and uvea of the mouse eye. Furthermore, EM showed that SCs were present in the anterior layer of the iris and limbus. Adhaerens and gap junctions were found to connect TCs within a network in uvea and sclera. Nanocontacts (electron-dense structures) were observed between TCs and other cells: SCs, melanocytes, nerve endings and macrophages. These intercellular ‘feet’ bridged the intercellular clefts (about 10 nm wide). Moreover, exosomes (extracellular vesicles with a diameter up to 100 nm) were delivered by TCs to other cells of the iris stroma. The ultrastructural nanocontacts of TCs with SCs and the TCs paracrine influence *via* exosomes in the epithelial and stromal SC niches suggest an important participation of TCs in eye regeneration.

## Introduction

The potential of stem cell (SC) therapies for eye diseases is well-recognized even the results remain only encouraging [1–7].

Recently, telocytes (TCs) have been described as a new type of interstitial cell by electron microscopy [8–10]. Telocytes have a small cell body and very long and thin cell prolongations (telopodes; Tp) with moniliform appearance, dichotomous branching and 3D-network distribution. Telocytes were found in close relationship with nerve endings, blood vessels and different types of resident cells, suggesting a role in the complex intercellular signalling throughout heterocellular junctions, shed vesicles and/or exosomes [11–14]. Particularly, TCs seem to be involved in the regenerative process because of their tandem with SCs in a variety of organs: heart [12, 15, 16], skeletal muscle [17], lung [18, 19], choroid plexus [20] or skin [21].

Adult tissue SCs are undifferentiated cells, capable of proliferation, self-renewal and differentiation into different tissue-specific progeny. Because of the functional definition, SCs are studied *in vitro* experiments and the microenvironmental interactions are not seen as integral part for their function [5]. Little is known about the cellular mechanisms responsible for eye renewal, regeneration and/or repair *in situ* but SCs and progenitor cells have been described in different areas of the eye [22–25]. Particularly, limbus seems to be rich in undifferentiated pluripotent cells which serve as an important source of new corneal epithelium [26–30] and a stem-cell niche has been described at this level [24].

We believe that it is critical to gain knowledge about the specific tissue environment where the stem/progenitor cells reside in eye. Therefore, we investigated the presence of TCs and SCs in mouse eye and their relationships using electron microscopy and electron tomography, as the appropriate diagnostic tools.

## Material and methods

Eyes from four C57BL/6 mice (12 months old) were used for the ultrastructural study after the Institutional Ethical Committee approval. Small samples of about 1 mm^3^ were fixed by immersion in 4% glutaraldehyde in 0.1 M cacodylate buffer, pH 7.4. Samples were post-fixed in 1% OsO_4_ with 1.5% K_4_Fe(CN)_6_ (potassium ferrocyanide reduced osmium) in 0.1 M cacodylate buffer. Samples were further dehydrated in increased graded of ethanol followed by propylene oxide and embedded in Epon [31]. Semi-thin sections (1 μm thick) were stained with 1% toluidine blue and examined by light microscopy (Nikon Eclipse E600, Tokyo, Japan).

### Transmission electron microscopy

Transmission electron microscopy (TEM) was performed on 60 nm thin sections stained with uranyl acetate and lead citrate using a Morgagni 268 electron microscope (FEI Company, Eindhoven, The Netherlands) at 80 kV. Digital electron micrographs were acquired with a MegaView III CCD and iTEM-SIS software (Olympus, Soft Imaging System GmbH, Münster, Germany). All measurements were performed with iTEM-SIS software, using 50 randomly selected structures/images.

### Electron microscope tomography

Electron microscope tomography (ET) was performed on 250-nm thick sections of Epon-embedded tissue [32] using a Tecnai G2 Spirit BioTwin transmission electron microscope with a single-tilt specimen holder (FEI Company) at 100 kV. Electron tomographic data sets were recorded with a MegaView G2 CCD camera (Olympus) in ET mode. Projection images (1024 × 1024 pixels) were acquired at 1-degree angular increments from −65 to +65 degrees around an axis perpendicular to the optical axis of the microscope, at 36,000× magnification (pixel size 2.65 nm). After data alignment, the data sets were reconstructed into 3D volume (data collection, reconstruction and visualization) using Xplore3D Tomography Suite software (FEI Company). Amira 5.0.1 software (Visage Imaging GmbH, Berlin, Germany) was used for 3D imaging.

## Results

**Fig. 1:**
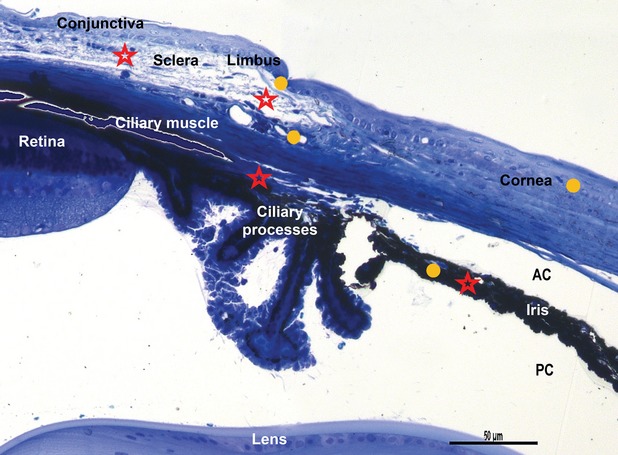
Light microscopy on semi-thin blue section of resin-embedded mouse eye. Stars indicated areas where telocytes are located: lamina propria of conjunctiva, limbal area, sclera, pars plana of the ciliary body, iris. Circles indicate the sites where stem cells have been found: cornea, limbus and iris. AC: anterior chamber; PC: posterior chamber. Toluidine blue staining, scale bar 50 μm, 20 × magnification.

**Fig. 2:**
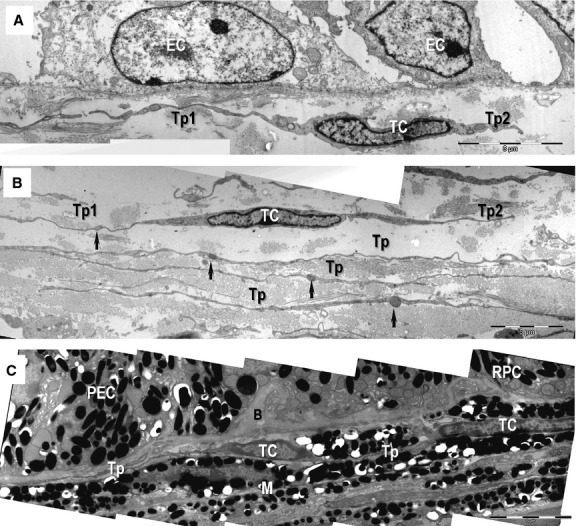
Transmission electron microscopy images show telocytes (TC) with telopodes (Tp) beneath the basement membrane of the conjunctival epithelium (**A**), sclera (**B**) and choroid (**C**). (**A**) Telocyte with two telopodes (Tp1, Tp2) beneath the corneal epithelium (EC) are visible. (**B**) Telocytes with overlapping telopodes (Tp) run in parallel layers in the sclera. The alternating thin segments (podomeres) and small dilations (podoms, arrows) generate the moniliform appearance of telopodes. (**C**) Telocytes extend telopodes (Tp) beneath Bruch's membrane (B) of the pigmentary cell of the retina (RPC) and ciliary body (PEC). Telopodes are more difficult to observe at lower magnification because of the electron-dense melanocytes (M); scale bars: **A**–**C** – 5 μm.

**Fig. 3:**
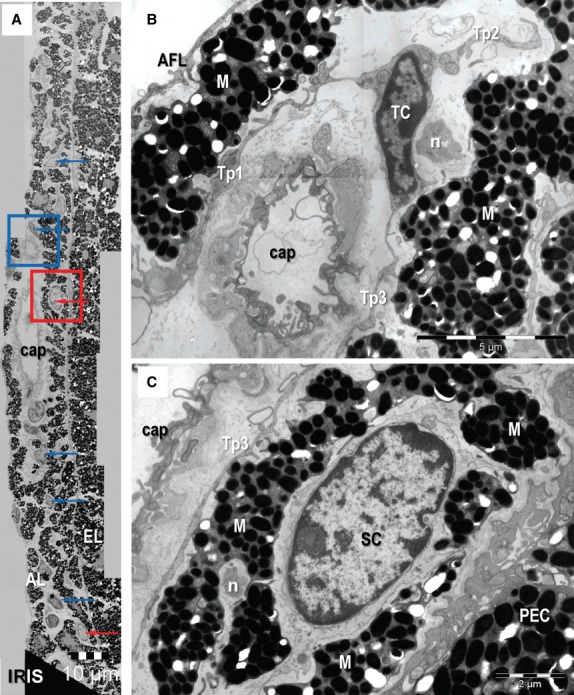
Transmission electron microscopy images of the mouse iris. (**A**) Telocytes (TC) (blue arrows) and putative stem cells (red arrows) are located between melanocytes in the anterior layer (AL). They are not present in the pigmented epithelial layer (EL) of the iris. Telocytes form a network in the stroma of the anterior marginal layer of the iris. (**B**) A higher magnification of the blue-square marked area in (**A**) shows a TC with three telopodes (Tp1-Tp3) extended among melanocytes (M), capillary (cap) and nerve endings (n). (**C**) Higher magnification of red square marked area in (**A**) shows a putative stem cell (SC) between M in the anterior layer of iris. PEC: pigmented epithelial cells of the iris. Scale bars: **A** – 10 μm; **B** – 5 μm; **C** – 2 μm.

**Fig. 4:**
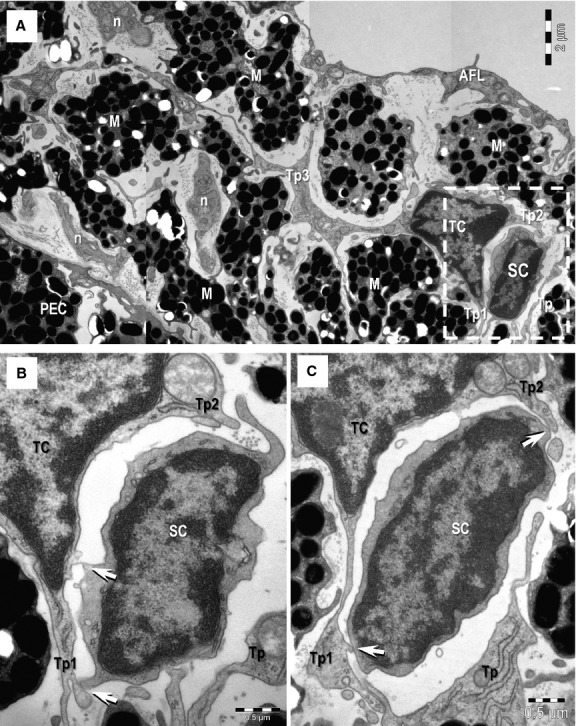
(**A**) Transmission electron microscopy image shows direct membrane-to-membrane contact (rectangular mark) between a telocyte (TC) and a putative stem cell (SC). M: melanocytes; n: nerves; AFL: anterior fibroblast layer. A TC with three telopodes (Tp1–Tp3) can be seen in the stroma of the iris. Tp3 extends between M and the dichotomous pattern of branching is noticeable. (**B**, **C**) Higher magnification of the TC-SC heterocellular connection (from rectangular marked area in **A**) – serial ultrathin sections. Tp2 and Tp3 enclose the putative SC. Small point contacts (arrows) connect the telocyte with the stem cell. Scale bars: **A** – 2 μm; **B**, **C** – 0.5 μm.

Ultrastructural analysis was performed on all three tunics of mouse eye (Fig. 1): fibrous (cornea and sclera), vascular pigmented (choroid, ciliary body and iris) and nervous (retina). Transmission electron microscopy showed the presence of interstitial cells with distinctive ultrastructural features defining TCs in lamina propria of the conjunctiva, in limbal area (Fig. 2A), sclera (Fig. 2B), beneath Bruch's membrane (Fig. 2C) and in the iris stroma (Figs 3A and B, 4A). TCs were not found in the ciliary processes, iris muscle, cornea or retina.

**Fig. 5:**
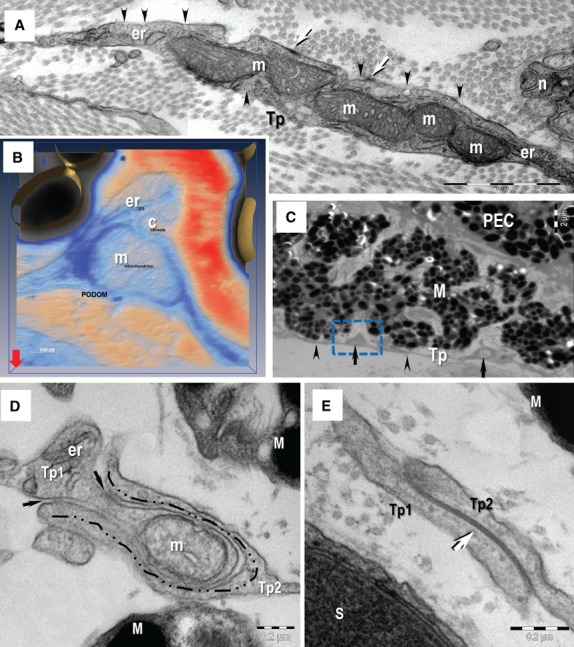
Transmission electron microscopy (TEM) image of the telopodes (Tp). (**A**) The Tp present small dilatation named podoms. The podoms accommodates mitochondria (m) and endoplasmic reticulum cisternae (er). Caveolae (arrowheads) and focal adhesion (arrows) are visible on the cellular membrane of telocyte at the podom level. n – nerve. (**B**) Electron tomography (3D isosurface reconstruction) of a podom illustrate the ‘Ca^2^^+^-uptake/release unit’ formed by mitochondrion (m), endoplasmic reticulum (er) and caveolae (c). (**C**) Rectangular mark indicates the podom on which electron tomography was performed on a thick section (200 nm). The Tp present alternating thin segments (podomeres, arrowheads) and small dilatation (podoms, arrows). (**D**, **E**) TEM images show different types of homocellular junctions connecting the telopodes (Tp1, Tp2): manubria adhaerentia (dashed line in **B**), puncta adhaerentia (black arrows in **B**) and gap junction (white arrow in **C**). M: melanocyte; S: Schwann cell; scale bars: **A** – 1 μm; **B** – 0.1 μm; **C** – 2 μm; **D**, **E** – 0.2 μm.

Telocytes showed an oval nucleus surrounded by a thin layer of cytoplasm (Figs 2A and B, 3B, 4A) and long cellular processes named Tp (Figs 2, 3B and 4A). Tp, very thin (below 100 nm) and long processes (up to 50 μm; 37.23 ± 9.72 μm), were the most prominent ultrastructural feature of TCs (Figs 8). The number of Tp per TC appears variable, usually two Tp in the sclera, choroid and ciliary body (Fig. 2) and three or more Tp in the iris stroma (Figs 3B and 4A). Tp showed characteristic uneven caliber and moniliform aspect (Fig. 2B) generated by alternating *podoms* (dilation of Tp, 294.47 ± 97.56 nm; Fig. 5) and *podomers* (slender segments; 95.67 ± 70.07 nm thickness; Figs 2 and 8). Podoms accommodate ‘Ca^2+^-*uptake/release units*’ formed by mitochondria, endoplasmic reticulum, caveolae (Fig. 5A and B).

**Fig. 6:**
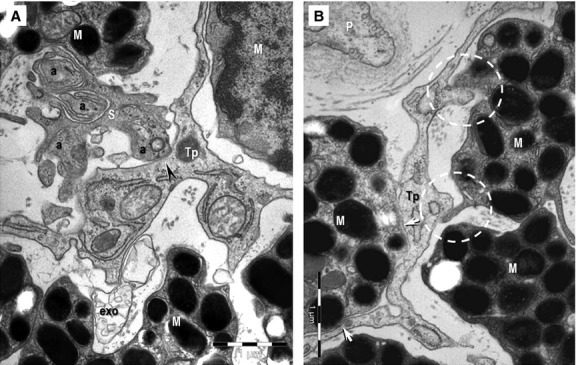
(**A**, **B**) Transmission electron microscopy images of the hetero-cellular connections formed by telocytes in the anterior layer of the iris. (**A**) Direct contact (arrowhead) can be seen between an axon (a) and a telopode (Tp). S: Schwann cell; exo: exosomes. (**B**) Point contacts (encircled) and planar contacts (arrows) are visible between a Tp and two melanocytes (M). P: pericyte.

**Fig. 7:**
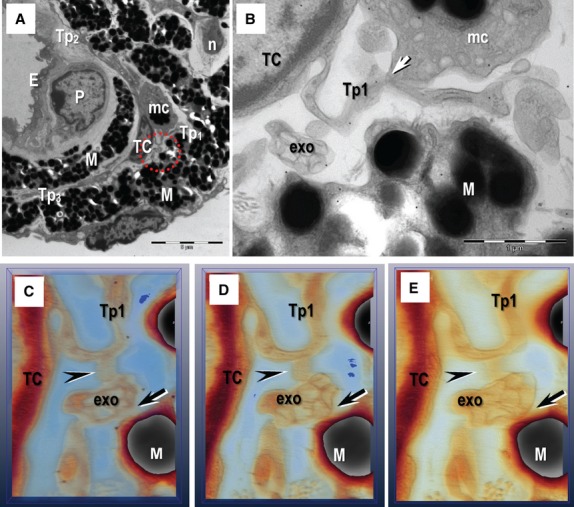
Electron tomography images on 200 nm thick section of resin-embedded iris. (**A**) General view shows a telocyte (TC) with three telopodes (Tp1–Tp3) in the anterior layer of iris. M: melanocyte; mc: macrophage; n: nerve; E: endothelial cell; P: pericyte. (**B**) Multi-vesicular structure containing exosomes (exo) is visible between a telocyte (telopode Tp1) and a M at higher magnification. Arrow indicates a point contact between telopode Tp1 and the mc. (**C**–**E**) Serial digital sections in the tomographic volume show that exo are connected with the Tp (arrowheads), as well as a M (arrows). Scale bars: **A** – 5 μm; **B** – 1 μm; **C**–**E** – 0.5 μm.

**Fig. 8:**
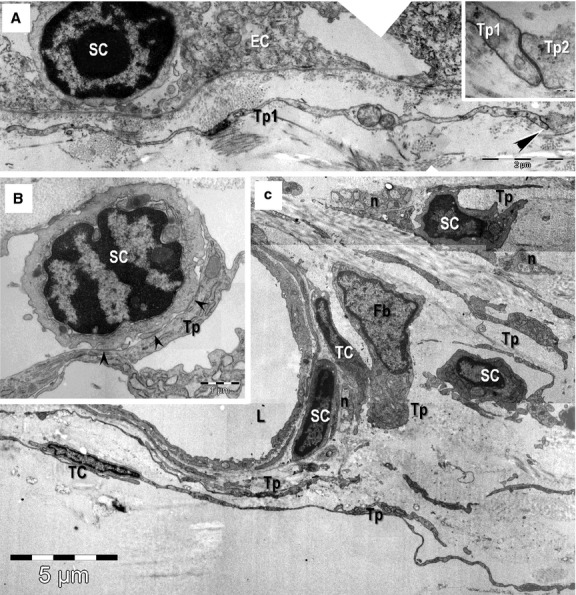
Transmission electron microscopy images of epithelial (**A**) and stromal (**B**, **C**) stem cell (SC) niches in the mouse eye. (**A**) Basal SC is sited on the basement membrane of limbus epithelium. A telopode (Tp1) runs parallel with the basement membrane and a gap junction (arrowheads) connect it with another one (Tp2; higher magnification in inset). (**B**, **C**) Stem cells in the stromal SC niches located in the corneoscleral junction. Direct contacts (arrowheads) between a Tp and the SC are visible in **B**. TC: telocytes; Tp: telopodes; Fb: fibroblast; n: nerve endings; L: lumen of an arteriole. Scale bars: **A** – 2 μm; inset – 0.1 μm; **B** – 1 μm; **C** – 5 μm.

Telocytes *via* Tp were connected in an interstitial network in sclera and uvea by different types of homocellular junctions (Fig. 5D and E): manubria adhaerentia, puncta adhaerentia, gap junctions or combination of these. In addition, non-canonical, heterocellular junctions connected TCs with stromal SCs (Figs 4 and 8B), nerve endings (Fig. 6A), melanocytes (Fig. 6B), and macrophages (Fig. 7A and B). These atypical junctions were characterized by direct membrane-to-membrane point contacts or planar contacts (Figs 4, 6 and 8B). The intercellular cleft was 10.99 ± 3.76 nm wide. Usually, about 10 nm small electron-dense nanostructures (feet) were observed bridging adjoining cellular membranes (Fig. 6). We did not found any connections between TCs and smooth muscle cells of pupillary and ciliary muscles or, between TCs and pigmented epithelial cells.

We often observed exosomes and shed vesicles (extracellular vesicles with a diameter below 100 nm) near TCs (Figs 6A and 7). Usually, an external membrane enclosed up to 10 exosomes near TCs (Figs 6 and 7C–E). Electron tomography showed that the exosomes were connected by point contacts with Tp and another type of cell, for example with melanocytes (Fig. 7) in the iris stroma.

Small cells (about 5 μm) with a high nucleo-cytoplasm ratio, containing few mitochondria, endoplasmic reticulum cisternae and numerous ribosomes in the scanty cytoplasm were found in the basal conjunctival epithelial layer at limbal level (Fig. 8A), in the stroma of the iris (Figs 3 and 4) and around blood vessels in the corneoscleral meshwork (Fig. 8B and C). Telocytes and SCs, alongside with nerve endings and blood vessels have been found as discrete clusters in these locations and have interpreted as stem-cell niches. Direct membrane-to-membrane contacts (nanocontacts or planar contacts) between TCs and SCs were often observed (Figs 4 and 8B).

## Discussion

Telocytes, as a novel type of interstitial cells, were characterized in details by electron microscopy [8, 10, 12, 13, 33, 34]. We report here the presence of TCs and SCs in limbus, sclera and uvea of mouse eye. Earlier electron microscope studies [35, 36] overlooked the existence of TCs at the level of sclera and uvea. In fact, TCs and SCs exist in the mouse eye alongside with melanocytes, pigmented epithelial cells, myoepithelial cells, smooth muscles cells, pigment-laden macrophages, fibroblasts, Schwann cells with nerve endings and capillaries. Moreover, TCs are interconnected in an interstitial network and are connected by ‘stromal synapses’ [37] with SCs, melanocytes, nerve endings, or macrophages.

Noteworthy, we found that TCs have contacts with SCs in discrete sites that seem to be stem-cell niches in the iris stroma and corneoscleral meshwork. The tandem TC-SC has been found in stem-cell niches in various organs (*e.g*. epicardium, lungs, skeletal muscle, choroid plexus, skin) [10]. Stem-cell niches are highly organized interactive structural units which commonly occur at tissue intersections or transition zones and coordinate tissue repair and renewal [24, 38–40]. The functionality of a stem-cell niche relies on the physical contact and signalling interactions of SCs with neighbouring nurse cells as well as the paracrine and endocrine signals from local or distant sources, neural input and metabolic products of tissue [38]. Telocytes seem to have ‘strategic’ positioning in the eye tissue, in between blood capillaries and their specific target cells (SCs, melanocytes, macrophages, *etc*.) and are in close contact with nerve ending. Telocytes could be nurse cells integrating local (short-distance signals: direct contacts, exosomes, shed vesicles) and long-distance signals through the long TPs, because of their 3D network in the eye stroma. Extracellular vesicles, exosomes and shed vesicles, participate in intercellular communications and seem to play key role in horizontal transfer of important bioactive macromolecules (*e.g*. membrane receptors, proteins, mRNAs) among neighbouring cells [41–44] and stem-cell niche [45]. Telocytes can even act in immune system modulation [46] or being ‘cellular’ guides for immune cells that arrive *via* blood stream [37]. On the basis of our observations, we agree with Cantarero *et al*. [33] supporting that TCs could be part of the ‘mesenchymal cell niche’ together with nerves fibres and blood vessels.

In addition, our study suggests that there are two different types of stem-cell niches into the eye: *epithelial niches* (basal cells in cornea and conjunctiva) and *stromal niches* (iris, corneoscleral junction). If in the epithelial niche, the contact of SCs with basement membrane seems to be prerequisite in the stromal niches the existence of stromal supporting cells (telocytes!?) is required [38, 39]. Recent studies show that spindle cells subjacent to limbal basal epithelial SCs serve as niche supporting cells which maintain clonal growth of limbal epithelial progenitors [47] possible by direct adhesion [48]. Present data show that TCs are neighbouring both epithelial and stromal SCs, but show (a)typical junctions [12] only with the stromal SCs (Fig. 8). It is required smart reparative cells to restore or repair or renew eye tissues, but it is also needed an architectural structure that keeps on the unit. And here is where TCs could play a nursing key role. The TCs network could even be a scaffold for SC migration between different layers of the eye.

Recent results showed a particular immunofenotype [9, 14, 19, 49], distinct microRNA expression [11, 50], specific gene-expression profile [51] and peculiar electrophysiological proprieties [52] of TCs in various organs. It remains to explore if all these proprieties are shared by eye TCs. Telocytes secrete VEGF and express platelet-derived growth factor receptor (PDGFR-β), both *in situ* and *in vitro* 10 in skeletal muscle [14] and border zone of myocardial infarction [11]. These finding suggest that TCs are an important player in promoting vasculogenesis. Their involvement in the pathophysiology of neovascular eye diseases should be investigated as antagonist of VEGF, and PDGF changed clinical practices for neovascular eye diseases [53–55].

## Conclusion

This ultrastructural study shows that TCs, coupled through adhaerens and gap junctions, form an interstitial network into the sclera and uvea creating a scaffold for SC migration between different layers of the eye. The tandem TC-SC present in eye stem-cell niches suggests that a heterocellular mixture could be more effective in the therapy of eye diseases.
